# Mpox in People Living with HIV: Clinical Challenges, Preventive Strategies and Public Health Implications

**DOI:** 10.3390/v17121558

**Published:** 2025-11-28

**Authors:** Rita Cordeiro, João Caria, Daniel Sobral, Diana Póvoas

**Affiliations:** 1 Emergency Response and Biopreparedness Unit, Infectious Diseases Department, National Institute of Health Doutor Ricardo Jorge, 1649-016 Lisbon, Portugal; 2 Institute of Environmental Health, Faculty of Medicine, University of Lisbon, 1649-028 Lisbon, Portugal; 3 Infectious Diseases Service, Local Health Unity Arrábida, 2910-549 Setúbal, Portugal; joaopedrocaria94@gmail.com; 4 Genomics and Bioinformatics Unit, National Institute of Health Doutor Ricardo Jorge, 1649-016 Lisbon, Portugal; daniel.sobral@insa.min-saude.pt; 5 Infectious Diseases Service, Curry Cabral Hospital, Local Health Unity São José, 1069-166 Lisbon, Portugal; diana.silva@ulssjose.min-saude.pt; 6 Gulbenkian Institute for Molecular Medicine, 2780-156 Oeiras, Portugal

**Keywords:** mpox, people living with HIV, co-infection, vaccination, antiviral therapy, public health

## Abstract

Monkeypox virus (MPXV) re-emerged in 2022 with a global outbreak that affected more than 100,000 individuals worldwide. People living with HIV (PLWH) accounted for a substantial proportion of cases, raising concerns about disease presentation, management, and outcomes in this population. Evidence indicates that PLWH with advanced or uncontrolled HIV infection experienced more severe mpox, with higher hospitalization rates, more complications, and longer disease courses. In contrast, individuals with well-controlled HIV generally had outcomes similar to those without HIV. Access to timely diagnosis, consistent antiretroviral therapy, and availability of tecovirimat were key factors influencing prognosis. Reports also suggest bidirectional interactions between mpox and HIV pathogenesis. Immune activation and APOBEC3-related viral evolution have been proposed; however, these mechanisms remain incompletely characterized and warrant further investigation. Moreover, disparities in healthcare access and stigma compound the vulnerability of PLWH, emphasizing the need for integrated approaches.

## 1. Introduction

Mpox, caused by the monkeypox virus (MPXV), is a zoonotic disease historically endemic to West and Central Africa, where human–animal interactions and wildlife reservoirs facilitated transmission. In recent years, however, mpox has emerged as a global health concern, highlighting its potential for international spread. Human-to-human transmission occurs mainly through direct contact with skin lesions, bodily fluids, or respiratory droplets from infected individuals or animals [1,2].

The global outbreak that began in May 2022 was unprecedented in scale and differed from earlier outbreaks by sustaining human-to-human transmission across international sexual networks. Most cases were reported among men who have sex with men (MSM), many of whom were people living with HIV (PLWH), underscoring shared transmission routes and specific immunological vulnerabilities in this population [3].

MPXV is currently classified into two major clades: clade I (Congo Basin, subdivided into Ia and Ib) and clade II (West African, subdivided into IIa and IIb). These clades differ in virulence, with clade I generally associated with more severe outcomes and higher case fatality rates [1,2,3]. The 2022 multinational outbreak was driven by clade IIb, which spread to more than 121 countries and led the World Health Organization (WHO) to declare a Public Health Emergency of International Concern, later lifted in 2023 [3]. Since then, clade Ib outbreaks have predominated in Central Africa, particularly in the Democratic Republic of the Congo (DRC), with growing evidence of international spread, and have progressively extended beyond the region. Recently, imported cases have been detected in multiple countries across Europe, Asia, and the Americas, including cases without travel history, indicating sustained human-to-human transmission outside Africa [3,4].

Epidemiological patterns also differ between clades. Clade IIb primarily affected MSM aged 30–39 years (87% of global cases), whereas clade I outbreaks in the DRC have disproportionately affected children under 15 years, who accounted for 70% of cases and nearly 90% of deaths [3,5,6,7]. Although global incidence declined after 2022, both clades continue to circulate, sustained by animal reservoirs and persistent human-to-human transmission [3]. The recent emergence and transcontinental spread of clade Ib underscore its potential for wider dissemination and highlight the urgent need for enhanced laboratory and genomic surveillance worldwide.

Mpox–HIV co-infection has become a pressing clinical concern. PLWH with advanced immunosuppression—particularly those with low CD4^+^ T-cell counts or uncontrolled viremia—face an elevated risk of severe disease, complications, prolonged viral shedding, hospitalization, and death. Conversely, individuals with well-controlled HIV generally present outcomes comparable to those without HIV. Mpox infection may also alter immune dynamics in PLWH, raising unresolved questions about antiviral efficacy, vaccine immunogenicity, and long-term management [8].

This review provides a comprehensive synthesis of the current evidence on mpox in PLWH, with emphasis on epidemiology, clinical features, pathogenesis, diagnosis, treatment, prevention, and public health implications.

## 2. Epidemiology of Mpox–HIV Co-Infection

During the 2022 outbreak, HIV co-infection emerged as a key determinant of disease burden and outcomes. WHO data indicated that most cases occurred among MSM (87.0%). Among those with known HIV status, 51.2% were people living with HIV (PLWH), a prevalence markedly higher than the background level in this group [3]. Multiple cohort studies across Europe and the Americas consistently reported 38–41% of mpox cases in PLWH, confirming the strong overlap with HIV-positive sexual networks [9,10,11].

Advanced immunosuppression was strongly linked to adverse outcomes. Hospitalization rates approached 30% among PLWH with CD4 counts <100 cells/µL, compared with 16% in those with CD4 >300 cells/µL. Mortality reached 27% in the most severely immunocompromised patients [8]. In African clade Ib epidemics, case fatality rates reached up to 10%, with children and PLWH disproportionately affected. Pediatric infections accounted for approximately 5–10% of reported cases in endemic regions, frequently linked to household transmission, whereas women represented less than 3% of global cases during the 2022 outbreak, though they remain particularly vulnerable in endemic settings through caregiving and community exposure rather than sexual transmission [8,12,13,14].

These findings underscore the critical need to integrate mpox surveillance, diagnosis, prevention, and treatment strategies within existing HIV care frameworks, particularly for populations at highest risk [3,15].

## 3. Clinical Manifestations

Before 2022, mpox typically presented with fever, malaise, lymphadenopathy, and a synchronous vesiculopustular rash. Most cases were self-limiting within 2–4 weeks, with case fatality rates (CFR) <4% for clade II and ~11% for clade I, particularly among children [9,16,17,18,19].

The 2022 multinational outbreak displayed distinctive features, including asynchronous lesion development, frequent genital and perianal involvement, and proctitis as an initial presentation, sometimes requiring hospitalization. Painful oral ulcers were common, whereas lymphadenopathy appeared less frequent than in earlier descriptions. Pregnancy was associated with poor outcomes, with fetal loss reported in up to 50% of cases [18,20,21,22].

Clinical expression varied by viral clade. In the 2024 DRC outbreak (clade Ib), children exhibited widespread extragenital lesions and higher morbidity, although overall mortality was relatively low (0.5%). However, pregnancy losses remained frequent among infected women [23]. By contrast, the global clade IIb epidemic predominantly affected MSM, with risk strongly linked to multiple sexual partners and concomitant sexually transmitted infections (STIs) [24].

Among PLWH, incidence ranged from 38 to 50% [21,25,26,27]. Genital, anal, and perianal lesions, proctitis, and severe necrotizing mucocutaneous disease were disproportionately observed, alongside systemic complications such as pneumonitis, central nervous system involvement, endocarditis, and ocular disease [21,25,26,27,28,29].

Systematic reviews and meta-analyses confirmed poorer outcomes in PLWH. Hospitalization risk was significantly higher (pooled RR 1.57; 95% CI: 1.18–2.08), particularly among those with CD4 counts <200 cells/µL (pooled OR 5.3; 95% CI: 2.0–14.1; *p* < 0.001) or unsuppressed viremia (pooled OR 3.0; 95% CI: 2.1–4.2) [26,30,31]. Mortality was also elevated (pooled OR 3.9; 95% CI: 2.27–6.65; *p* < 0.001), with most deaths occurring in severely immunosuppressed individuals [26,29,31,32]. Fatality rates up to 13% were reported among hospitalized cases [26,27].

Collectively, these findings support proposals to classify disseminated or necrotizing mpox as an AIDS-defining condition [8,28,33].

## 4. Diagnosis and Laboratory Considerations

Timely diagnosis of mpox is critical, particularly in PLWH with immunosuppression, who face a higher risk of severe disease. In suspected cases, especially where sexual transmission is possible or HIV status is unknown, the WHO recommends concurrent HIV testing alongside mpox evaluation [21,34].

Nucleic acid amplification testing (NAAT), particularly real-time PCR, remains the diagnostic gold standard. Assays can either detect *Orthopoxvirus* broadly or MPXV specifically, while clade-specific PCR and whole-genome sequencing (WGS) may further refine diagnosis and inform surveillance [35]. Lesion swabs and crusts are the preferred samples; oropharyngeal swabs can also be informative. Depending on clinical presentation, anorectal swabs, semen, urine, cerebrospinal fluid, or vitreous fluid may be collected [35]. PCR testing of EDTA whole blood has limited sensitivity due to the brief duration of viremia. Serology is not routinely recommended, although acute IgM detection or a four-fold rise in IgG titers may support diagnosis [35].

Basic laboratory investigations should include complete blood count, renal and hepatic function tests, and cultures for bacterial superinfection. Reported abnormalities include leukocytosis, elevated transaminases, altered blood urea nitrogen (low or high), hypoalbuminemia, and cytopenia [21,34,35,36].

The differential diagnosis is broad and includes varicella-zoster virus (VZV), herpes simplex virus (HSV), syphilis, disseminated gonococcal infection, measles, scabies, dengue, Zika virus, vasculitis, and bacterial skin infections. In immunocompromised patients, disseminated cryptococcosis may also mimic mpox [21,35]. Unlike VZV, mpox typically shows slower lesion progression, a more centrifugal rash, and lymphadenopathy, which is uncommon in varicella. Lesions on palms and soles may occur, but are not consistent features. PCR-confirmed mpox–VZV co-infection has been reported in the Democratic Republic of the Congo (10–13%), presenting with overlapping clinical signs and an intermediate lesion burden between both infections. Co-infections with other STIs, such as syphilis or gonorrhea, are also frequent in sexual transmission networks [21].

Hospitalization is strongly recommended for PLWH with CD4 counts <100 cells/µL or uncontrolled HIV viremia, due to the high risk of necrotizing lesions, bacterial sepsis, and systemic complications [8,35]. Importantly, while diagnosis and laboratory work-up are similar for individuals with and without HIV, the threshold for hospitalization and the intensity of monitoring must be lower in PLWH, particularly those with advanced immunosuppression.

## 5. Intra-Host Genetic Variability in PLWH

**Figure 1:**
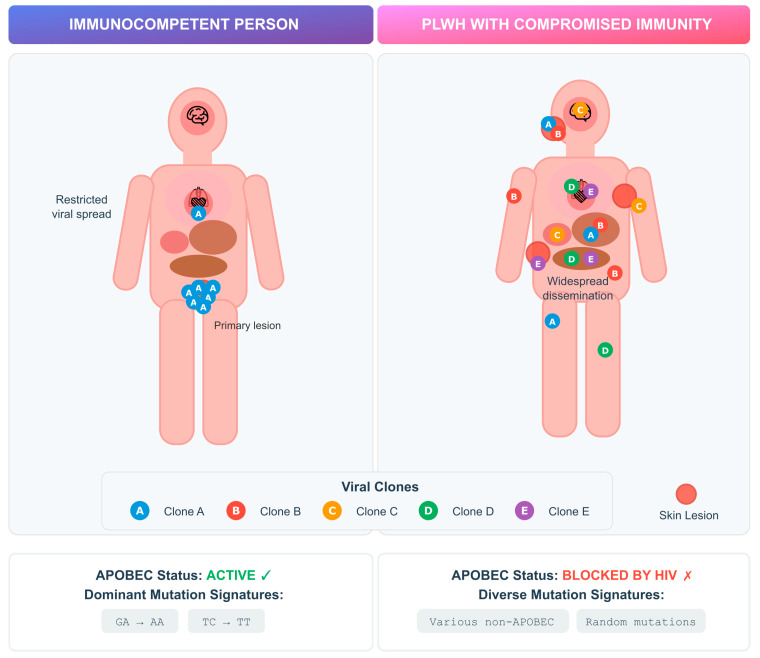
Intra-host MPXV genetic variability in PLWH versus immunocompetent individuals. Schematic representation of viral replication dynamics and mutational patterns according to host immune status. In immunocompetent individuals (**left**), APOBEC3-mediated cytidine deamination restricts viral replication and drives characteristic G→A and TC→TT mutation signatures. In PLWH with advanced immunosuppression (**right**), impaired APOBEC activity permits widespread viral dissemination and intra-host diversification, resulting in multiple viral clones and heterogeneous, non-APOBEC mutation patterns.

Advanced HIV infection provides a permissive environment for persistent MPXV replication, which may drive intra-host diversification. Similar dynamics have been observed in other viral infections, such as SARS-CoV-2, where prolonged replication in immunocompromised hosts enabled rapid viral evolution [37] (Figure 1).

Case studies have described multiple viral clones in single mpox infections, including in PLWH, suggesting diversification can occur early in infection [38,39]. MPXV, as a DNA virus, mutates more slowly than RNA viruses, and much of its diversity is thought to result from APOBEC-mediated editing [40,41]. APOBEC (apolipoprotein B mRNA-editing enzyme catalytic polypeptide-like) proteins are host cytidine deaminases that normally restrict viral replication by inducing G-to-A hypermutations in viral genomes. In MPXV, APOBEC activity leaves a characteristic mutational signature likely to have been an important driver of viral evolution during the 2022 outbreak, as well as suggesting sustained human transmission since at least 2016 [40,41]. Interestingly, studies in patients with advanced HIV revealed novel mutations lacking the characteristic APOBEC signature. In particular, a Vietnamese cohort study involving patients with severely compromised immune systems (median CD4 count of 14/µL) revealed 10/12 new mutations without APOBEC patterns, suggesting alternative evolutionary mechanisms under immunosuppression [42].

Comparative analyses further highlight divergent dynamics depending on immune status. An Italian study identified that in immunocompetent patients, identical viral clones were usually detected across anatomical sites, whereas in advanced HIV, prolonged infection allowed distinct clones to emerge in different tissues over time [43]. This compartmentalized evolution may affect transmissibility, shedding duration, and treatment outcomes.

The relatively low mutation rate of MPXV makes it hard to distinguish between within-host evolution and simultaneous infection [44]. Although co-infections with multiple strains have been reported, most observed diversity likely reflects within-host evolution rather than simultaneous infection by different lineages [44,45]. Moreover, technical limitations may also be hindering our ability to detect viral diversity. Beyond point mutations, MPXV exhibits genome plasticity through structural changes such as gene duplication, loss, and ITR expansion, which may enhance adaptability but are often missed in routine sequencing [44].

Despite HIV being disproportionately represented among mpox cases, studies of viral genomic diversity from PLWH remain scarce, especially from Africa, where both HIV and MPXV are endemic and clade I outbreaks continue to drive high morbidity [46]. Recent sequencing efforts are beginning to bridge this gap, but more studies integrating longitudinal, multi-site sampling with immunological and clinical data are needed. Single-cell transcriptomic analyses suggest that even virologically suppressed PLWH display distinct immune responses during acute mpox, potentially influencing viral diversification [47].

In sum, intra-host MPXV variability in PLWH appears to result from prolonged replication, impaired immune control, and tissue-specific pressures. Understanding these dynamics will require dedicated studies and enhanced genomic surveillance, particularly in endemic regions.

## 6. Immunopathogenesis and Disease Progression

### 6.1. Viral Load Dynamics During Mpox Infection

**Figure 2:**
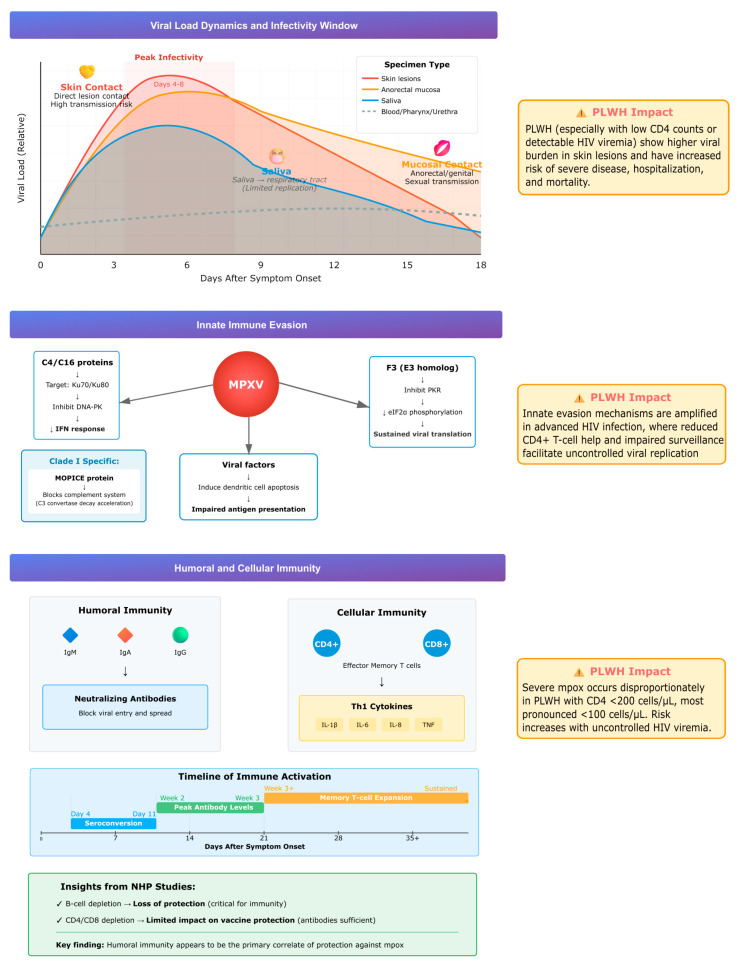
Summary of viral load dynamics, immune evasion, and host responses during mpox infection, highlighting higher viral burden and impaired immunity in PLWH.

Understanding viral load dynamics and viable virus shedding in different types of clinical lesions has been shown to be relevant to patient care, severity assessment and infection control (Figure 2). PLWH tend to present higher viral loads in skin lesions, and reduced CD4^+^ T-cell counts or uncontrolled HIV viremia correlate with severe disease, hospitalization, and mortality. A systematic review and meta-analysis of 19 studies (880 patients, 1477 specimens) confirmed that skin lesions, anorectal swabs, and saliva contain the highest viral DNA, while pharyngeal, urethral, and blood samples usually show lower levels. Anorectal and saliva specimens also yielded viable virus more frequently, supporting their role in transmission. Viral loads typically peaked 4–8 days after symptom onset, with viable virus persisting for 14–19 days depending on specimen type. Although saliva contained high viral loads, the respiratory tract does not appear to be the primary replication site, suggesting that direct mucosal contact rather than inhalation plays a larger role in transmission. PLWH showed significantly higher viral burden in skin lesions, though differences in other sample types were less pronounced [48]. Taken together, these data support that the peak infectious period takes place early in the disease course and may persist for up to three weeks, highlighting the importance of early detection and infection prevention measures to be implemented. Clinical monitoring is of particular relevance in the setting of uncontrolled HIV infection, due to higher viral loads and worse outcomes in the setting of immunocompromise.

### 6.2. Innate Immunity Evasion

MPXV employs several strategies to evade host innate immunity. Viral proteins C4 and C16 inhibit DNA sensing by targeting the Ku heterodimer (Ku70/Ku80), which normally activates the DNA-dependent protein kinase (DNA-PK) pathway, a key sensor of cytosolic DNA that triggers interferon responses. By blocking this pathway, MPXV prevents recognition of its genome as a danger signal. The virus also suppresses natural killer (NK) cell function by reducing TNF-α and IFN-γ secretion. The F3 protein, a vaccinia virus E3 homolog, disrupts the interferon–protein kinase R (IFN–PKR) axis by blocking PKR activation and eIF2α phosphorylation, thereby suppressing interferon signaling and sustaining viral protein synthesis. In dendritic cells, MPXV induces apoptosis, impairing antigen presentation and adaptive immune priming [49].

A distinctive virulence factor of clade I (Congo Basin) strains is the MPXV inhibitor of complement enzymes (MOPICE), which binds complement proteins and accelerates C3 convertase decay, blocking both classical and alternative complement pathways. This complement evasion likely contributes to the greater virulence and higher case fatality rates associated with clade I compared with clade II [49].

These mechanisms operate in all hosts but are particularly consequential in PLWH with advanced immunosuppression, where diminished CD4^+^ T-cell function and impaired immune surveillance exacerbate viral escape and promote severe, uncontrolled infection.

### 6.3. Humoral and Cellular Immunity

Acute MPXV infection induces rapid and effective immune responses. Early seroconversion with IgM, IgA, and IgG occurs, along with neutralizing antibody development [50]. Infection also triggers expansion of effector memory CD4^+^ and CD8^+^ T cells, accompanied by a Th1-skewed cytokine profile characterized by elevated IL-1β, IL-6, IL-8, and TNF [51,52]. Protection against severe disease in non-human primate models of vaccination appears primarily antibody-mediated, as B-cell depletion abrogates protection, whereas depletion of CD4^+^ or CD8^+^ T cells has a limited effect [51]. This highlights the central role of humoral immunity, although cellular immunity contributes to viral clearance. In PLWH, progressive CD4^+^ T-cell depletion compromises both arms of the immune response. Clinical data confirm that PLWH with CD4 counts <200 cells/µL, and especially <100 cells/µL, are disproportionately affected by severe mpox. In a series of 382 HIV-positive patients, lower CD4 counts were strongly associated with disease severity compared with those maintaining CD4 counts of 200–350 cells/µL [8].

### 6.4. Immune Humoral Response to Infection in Vaccinated Patients

Seroconversion for IgG typically occurs between days four and seven after symptom onset (median 7.5 days), while IgM/IgA appear between days eight and 11. Antibody titers rise progressively, peaking during the second and third weeks. Neutralizing antibodies become detectable around one week after symptom onset and remain stable for at least 20 days. In a study, IgG/IgM titers in patients treated with antivirals seemed to transiently decline between days 12–15, possibly due to reduced antigen load, though this requires further research. Humoral immune responses do not seem to be significantly altered by HIV status or prior smallpox vaccination [50].

Additional studies indicated that prior smallpox vaccination and mpox infection induce cross-reactive antibodies, with enhanced neutralizing capacity observed in individuals with hybrid immunity. Seroneutralization assays further demonstrated that complement activity augments neutralization against both MVA and MPXV [51].

Large serological analyses including individuals with and without HIV showed comparable antibody levels against vaccinia (A27L, A33R) and mpox (A29L, A35R) antigens among those born before 1981, consistent with waning immunity following cessation of universal smallpox vaccination [53].

Overall, the interplay between viral load dynamics, immune evasion, and host responses determines disease progression, as illustrated in Figure 2.

## 7. Antiviral Treatment and HIV-Specific Considerations

The 2022 clade II outbreak prompted unprecedented efforts to identify effective therapies for mpox, historically a neglected disease. Tecovirimat (TPOXX, ST-246) rapidly became the frontline antiviral, supported by animal studies showing survival benefit. Expanded access programs and trials such as PALM007 (DRC), STOMP (multicountry), and UNITY (multicountry) confirmed safety but showed no significant reduction in time to lesion healing or complications [54,55,56]. Importantly, these studies excluded or did not stratify outcomes for PLWH, leaving efficacy in this group uncertain.

In real-world settings, more than 7000 patients in the United States (U.S.) received tecovirimat under expanded access, approximately 52% of whom were PLWH. Adverse events were rare, but efficacy data remain limited [57]. In a 2024 cohort from Atlanta (*n* = 112 PLWH) early treatment group (<seven days) had reduced progression to severe disease compared with later initiation (5.4% vs. 26.8%), although these findings are based on limited data and should be interpreted with caution. However, tecovirimat has a low barrier to resistance, with cases reported of resistance after prolonged therapy (>two weeks) [58,59].

Alternative antivirals include cidofovir, previously used for CMV retinitis, though its application in severe mpox has been limited by nephrotoxicity and the need for intravenous hydration with probenecid co-administration [60,61]. Brincidofovir (also known as CMX001 or Tembexa), a cidofovir prodrug with reduced renal but increased hepatic toxicity, shows activity against orthopoxviruses in vitro and animal models. Limited case series reports mixed outcomes, and the MOSA trial (DRC, 2025) is ongoing [62]. Vaccinia immunoglobulin (VIGIV), licensed for vaccinia complications, has been used under expanded access in severe mpox with good tolerability but unproven efficacy [63,64].

Trifluridine, an ophthalmic antiviral, shows activity against mpox and tecovirimat-resistant strains, with case reports supporting its use in ocular disease, though corneal toxicity is a concern [65,66,67].

A comparative summary of major infectious disease society guidelines on antiviral treatment for mpox, including HIV-specific considerations, is provided in Table 1.

## 8. Vaccination and Preventive Strategies in PLWH

Vaccination is central to mpox prevention, particularly for vulnerable groups such as PLWH. Current strategies rely on vaccines originally developed for smallpox. First- and second-generation vaccines (e.g., Dryvax^®^, ACAM2000^®^) used live replicating vaccinia virus and carried risks of severe adverse events [70]. Third-generation vaccines, particularly MVA-BN (Imvanex^®^ in Europe, JYNNEOS™ in the U.S., IMVAMUNE^®^ in Canada), contain non-replicating modified vaccinia Ankara and have a favorable safety profile [71,72,73,74]. Regional alternatives include C16-KMB (Japan) and OrthopoxVac (Russia) [75].

Evidence for efficacy is extrapolated from smallpox vaccination (~85% protection) and human and nonhuman primate studies [76,77,78,79,80]. Clinical trials involving >5000 naïve participants confirmed good safety, with fewer adverse events in MVA-BN compared to the ACAM2000^®^ [81]. During the 2022 outbreak, intradermal administration (0.1 mL) was adopted to address shortages, showing comparable immunogenicity to the standard subcutaneous route (0.5 mL) [82,83].

For PLWH, data are encouraging. A U.S. phase II trial showed 80% seroconversion after one dose in antiretroviral therapy (ART)-treated PLWH (CD4 200–750/µL) [84]. Observational studies indicate reduced protection after a single dose of MVA-BN (vaccine effectiveness (VE) ~35% vs. 84% in HIV-negative participants), but robust protection with two doses, with no breakthrough infections reported in fully vaccinated PLWH in one study [85]. Meta-analyses confirm VE of 35–86% and 66–90% after one and two doses, respectively, with outcomes in PLWH broadly comparable to the general population when fully vaccinated [86].

Breakthrough infections and reinfections have been reported both in people with and without HIV infection, typically milder than primary infections, with lower hospitalization and fewer mucosal lesions [87]. Most occurred 6–9 months after vaccination, suggesting waning immunity [88]. Animal and human studies support consideration of booster doses at 2–3 years after vaccination, particularly for high-risk groups such as MSM with multiple partners, sex workers, and travelers to endemic regions [88,89,90,91].

Vaccine uptake and willingness remain challenges. While willingness among MSM seems to be as high as 77% in some studies, it may show variability in different settings that provide care for PLWH, influenced by personal concerns about vaccine safety, perception of risk, access to health services, engagement in high-risk sexual exposure, mpox risk awareness and knowledge about the [92]. Real-world uptake appears to be lower, with a systematic review estimating only 35.7% among PLWH [93]. Of note, during the 2022 outbreak, mpox prevention strategies were hindered by vaccine shortage and available vaccines, until very recently, were not available in many African countries. Addressing stigma, misinformation, and access barriers is essential to maximize protection.

Beyond vaccination, innovative preventive strategies such as wastewater surveillance and community-based interventions (e.g., partnerships with Non-Governmental Organizations, dating apps, and venues) have proven valuable in early detection and targeted risk communication [94,95,96,97].

## 9. Public Health Implications

The overlapping epidemiology of mpox and HIV underscores the need for integrated approaches to surveillance, prevention, and care. PLWH, particularly those with advanced immunosuppression, face a disproportionate burden of severe disease, hospitalization, and mortality, making them a priority group for clinical and public health interventions.

Early recognition and prompt diagnosis are critical to mitigate severe outcomes, especially in immunocompromised patients who may present atypically. Integration of HIV and mpox services, including testing, vaccination, antiviral access, and follow-up, can improve outcomes and reduce delays in care [34].

At the prevention level, equitable access to vaccination is essential. While MVA-BN has demonstrated safety and immunogenicity in PLWH, reduced effectiveness after a single dose highlights the importance of completing two-dose schedules and considering boosters in high-risk groups [85]. Addressing barriers such as stigma, misinformation, and structural inequities will be key to improving vaccine uptake and trust among vulnerable populations.

Genomic surveillance must also be strengthened, particularly in endemic regions of Africa where HIV and mpox co-circulate and clade I outbreaks continue to drive high morbidity and mortality. Enhanced sequencing, coupled with clinical and immunological data, will be vital to monitor viral evolution in PLWH and to inform treatment and prevention strategies. Complementary tools, such as wastewater monitoring and community-based digital health interventions, can support early outbreak detection and targeted communication [94,95,96,97].

## 10. Conclusions

Mpox has re-emerged as a global health concern with profound implications for PLWH. Evidence consistently shows that advanced immunosuppression is associated with more severe disease, prolonged viral shedding, and increased risk of hospitalization and death. Antiviral options remain limited, with tecovirimat widely used but lacking robust efficacy data in PLWH, while other therapies require further evaluation. MVA-BN vaccination is safe and immunogenic in this population, but full schedules and, potentially, booster doses are needed to ensure adequate protection.

Addressing the dual burden of mpox and HIV requires integrated prevention and care strategies, equitable access to antivirals and vaccines, and strengthened surveillance, particularly in endemic regions. Including PLWH in clinical trials and genomic studies is essential to close current knowledge gaps. A comprehensive, equity-driven response is critical to reduce disparities in disease outcomes and to improve preparedness for future *Orthopoxvirus* threats.

## Figures and Tables

**Table 1:** Summary of treatment recommendations in mpox [54,68,69].

Source	Supportive Care	First-Line Antiviral (Tecovirimat)	Second-Line/Alternatives	Other Considerations
[54]	Supportive care for mild disease in well-controlled HIV.	Recommended for severe mpox or high-risk PLWH (CD4 < 200/µL). Early initiation advised.	Cidofovir, brincidofovir, or VIGIV for severe/refractory disease. Topical trifluridine for ocular involvement.	ART initiation in untreated patients. Consider prolonged treatment (>14 days) if no improvement.
[68]	Supportive care for mild–moderate disease and in immunosuppressed. Pain control, hydration, and management of skin lesions.	Recommended for severe, immunocompromised, or complicated cases.	Cidofovir, brincidofovir, or VIGIV for severe/refractory disease.	–
[69]	Supportive care for mild illness. Pain control, hydration, management of skin lesions.	Tecovirimat recommended for severe disease or immunocompromised PLWH. Oral/IV formulations available.	Consider cidofovir, brincidofovir, VIGIV for severe/refractory disease; topical trifluridine for ocular involvement.	Multidisciplinary care recommended for severe disease in advanced HIV.

## Data Availability

No new data were created or analyzed in this study.

## Abbreviations

The following abbreviations are used in this manuscript:

AIDS	Acquired immune deficiency syndrome
APOBEC	Apolipoprotein B mRNA Editing Catalytic Polypeptide-like
ART	Antiretroviral Therapy
CD4	Cluster of Differentiation 4
CDC	U.S. Centers for Disease Control and Prevention
CFR	Case fatality rate
CI	Confidence interval
CMV	Cytomegalovirus
Ct	Cycle thresholds
DRC	Democratic Republic of the Congo
EDTA	Ethylenediaminetetraacetic Acid
HIV	Human immunodeficiency virus
HSV	Herpes Simplex Virus
IgG	Immunoglobulin G
IgM	Immunoglobulin M
ITR	Inverted terminal repeats
MPXV	monkeypox virus
MSM	Men who have sex with men
NAAT	Nucleic acid amplification testing
NIH OI	U.S. National Institutes of Health Opportunistic Infections
NYSDOH	New York State Department of Health
OR	Odds ratio
PCR	Polymerase Chain Reaction
PLWH	People living with HIV
RR	Relative risk
STIs	Sexually transmitted infections
US	United States
VIGIV	Vaccinia Immune Globulin Intravenous
VZV	Varicella-Zoster Virus
WGS	Whole-genome sequencing
WHO	World Health Organization
